# Clinical learning evaluation questionnaire: a reliable and valid tool for the evaluation of clinical education by educators and students

**DOI:** 10.1186/s12909-023-04601-w

**Published:** 2023-08-30

**Authors:** Jeyran Ostovarfar, Saeed Kazemi Soufi, Mahsa Moosavi, Somayeh Delavari, Mana Moghadami, Seyed Mehdi Ghazanfari, Mitra Amini

**Affiliations:** 1 MPH Department, Medical School, Shiraz University of Medical Sciences, Shiraz, Iran; 2 Shiraz University of Medical Sciences, Shiraz, Iran; 3 Clinical Education Research Center, Shiraz University of Medical Sciences, Shiraz, Iran; 4 Center for Educational Research in Medical Sciences (CERMS), Department of Medical Education, School of Medicine, Iran University of Medical Sciences, Tehran, Iran

**Keywords:** Reliability, Validity, Clinical learning, Medical students

## Abstract

**Background:**

The clinical learning evaluation questionnaire can be used in the clinical trial period of medical students to measure the effectiveness of the clinical learning environment. The purpose of this study was to collect validity evidence of an adapted questionnaire to measure the transcultural adaptation of a Persian version of CLEQ.

**Methods:**

A total of 200 questionnaires were completed by students who were at the end of their clinical rotation. The study instrument was the latest version of the CLEQ consists of 18 Items in four dimensions. The CLEQ was translated into Persian language through a four-step process of forward and backward translation. Data analysis was performed on two softwares, SPSS, version 22 and Lisrel, version 8.8.

**Results:**

The results showed that the 18-question CLEQ could be applied to the Persian translation of the tool. The response process evidence of the Persian questionnaire was established through feedback from 15 students in the sample group. The content validity index (CVI) for the items were between 0.8 and 0.9, and the content validity ratio (CVR) for the entire questionnaire was 0.9. The 4-factor feature of CLEQ was good model fit. The internal consistency analysis indicated that the Cronbach's alpha values for all items of the 18-item questionnaire were equal to 0.87 and for the subscales were 0.68 to 0.79.

**Conclusion:**

The Persian translation of the 4-factor CLEQ has sufficient validity evidence to measure the transcultural adaptability of clinical education activities by instructors and students. The validity evidence are content, response process and internal structure. We recommend that the English 6-factor and 6-factor versions of CLEQ be tested on medical students at multiple foreign academic institutions to assess their efficiency.

## Introduction

Training competent medical professionals on the management and problem solving of common medical issues is the most critical purpose of medical education [1]. The learning environment is the most critical setting for undergraduate and postgraduate medical students, because it combines learning and clinical practice in a dynamic context [2]. Such an environment is a critical component for the learners and their educational experiences [2]. Also, it is a crucial determinant of their clinical behavior and is related to the learners' achievements, success, and professional satisfaction [3]. The interactions between the learning environment and learners can have both positive and negative effects. The positive effect includes enabling the learner to succeed, and provide high-quality care to patients. The negative effect includes burnout, low-quality patient care, and mis-learning issues [2, 4].

Clinical rotations are of vital importance; they provide medical students with various clinical situations during their training in medicine [1]. They ensure the effectiveness of students’ learning and teaching during the rotations [1]. Therefore, accreditation agencies and medical schools have placed more emphasis on assessing their learning environment. These agencies encourage medical schools and teaching hospitals to explore appropriate manners to assess and monitor their learning environment [2]. Obviously, learning is a complex process at both inpatient and outpatient settings. Many factors, such as the quality of feedback, length of time spent with patients in the clinical setting, exposure to various medical conditions, and the quality of supervision by the preceptors could affect the learning quality [1, 5]. Therefore, these factors, among others, challenge and implicate the evaluation of the clinical environment for both the students and faculty [6].

There are various instruments for the evaluation of the educational environment at different settings [1]. Two tools that are frequently used in medical education include Postgraduate Hospital Educational Environment Measure (PHEEM) [7] and Dundee Ready Education Environment Measure (DREEM) [8]. These tools are used for assessing the overall quality of learning environment and its effect on the actual learning process [1]. The focus of PHEEM and DREEM is on the academic facilities, atmosphere, and psychosocial characteristics of the learning environment [1]. Another similar tool is Clinical Learning Evaluation Questionnaire (CLEQ) [1, 9, 10]. In contrast to other tools, CLEQ has been developed based on the factors that contribute to effective clinical learning [11]. It is inclusive of six different factors, such as cases, authenticity, supervision, organization, motivation, and self-awareness [1]. In 2020, an study by Nuha Alnaami, et al. did not support the 6-factor structure and showed that the 4-factor structure of CLEQ, i.e., cases, organization, supervision, and motivation, has a sufficient degree of good fit and is as reliable as the original version [12]. As a result, it seems that more research is needed to predict the validity of CLEQ with 18 questions, and compare the psychometric properties in other languages. In addition, there is a need for a valid and reliable Persian version of CLEQ to assess the condition of the clinical environment in the Iranian teaching hospitals.

### Aim of the study

Given the above facts, this study was conducted to collect valid evident of an adapted questionnaire to measure the transcultural adaptation of a Persian version of CLEQ by Iranian educators and students.

## Materials and methods

### Sample size calculation and participant recruitment

The sample size was obtained from the N/p ratio, that is, the ratio of item to participant should be at least 1/10, which represents 10 respondents for each item in the questionnaire [13]. Therefore, the 18-question questionnaire required a sample size of 180 participants. Sampling method was random sampling.Considering a 10% dropout, 200 students were recruited. Participating students entered the study at the end of their clinical rotation because they seemed to be better able to express their views on various aspects of the clinical learning environment. The students were from the clinical wards of the hospitals under the supervision of Shiraz University of Medical Sciences, including Chamran, Namazi, Shahid Faghihi and Hafez hospitals.

Before starting the study, ethical clearance was obtained from the Shiraz University of Medical Sciences. Before initiating research activities, informed consent from participants was obtained. The confidentiality and anonymity of the data were guaranteed. We also informed the participants of their right to refuse to participate for any reason without penalty.Medical interns excluded from the research that were not fluent in Persian or did not, or they did not want to complete the questionnaire.

### Data gathering tool and procedure

In this study, we used the Persian version of CLEQ to respond to the study's purpose in the first section. The CLEQ, as designed by AlHaqwi, Kuntze, and Molen contains a total of 40 items [14]. However, based on the results of Nuha Alnaami et al., the latest version of the CLEQ questionnaire, which had a 4-factor structure with 18 items, was studied [12]. The numbers of the questions in the figures are numbered based on the 40 primary questions. The CLEQ questionnaire evaluate 4 main areas that effect on students' clinical learning that include: provision of clinical cases (4 items), organization of the doctor-patient encounters (5 items), supervision (4 items), and motivation of students to learn (5 items). The response for each item is given through a 5-point Likert scale. For the questions, scores of 5, 4, 3, 2, and 1 are assignedto “strongly agree”, “agree”, “undecided,”, “disagree”, and “strongly disagree”, respectively [14].

### Persian translation of CLEQ

According to the four consecutive stages of translation and back translation proposed by Chen et al., the CLEQ was translated [15]. During the translation, the translators emphasized conceptual accuracy rather than verbal accuracy and paid attention to an acceptable linguistic approach for Persian-speaking participants. To conduct the study, after obtaining the consent of the authors of this questionnaire, first the original version of the questionnaire was translated into Persian language by two people who speak Persian and who are fluent in English. One of the translators was fluent in medical education and the other translator was independent translator for whom English was the mother tongue and who had no knowledge related to the questionnaire. Agreements on differences in translation were reached through discussion between two translators, and the final version of the Persian questionnaire was prepared. Then, to eliminate the conceptual inconsistency, the Persian version of the questionnaire was translated into English by two native English speaking translators. The translators carried out the process of translation and back translation in coordination with the researcher.

### Content validity and response process

The final version of the Persian questionnaire was given to 15 students from the same sample group who did not participate in the main study to check the response process evidence of the questionnaire and eliminate possible problems. The results of these steps were reviewed by the researchers and the final comments were included by the translators in the final version of the Persian version of CLEQ.

The Persian version of the CLEQ questionnaire was checked to determine the response process evidence of the questions in terms of writing style, clarity and fluency. Therefore, content validity index (CVI) and content validity ratio (CVR) were used to ensure the accurate and conceptual matching of the original CLEQ questionnaire and the Persian version. Therefore, to check the content validity, 10 experts were hired to answer each question of the questionnaire to check the essentiality of the questions based on Lawshe’s study [16].

To check the necessity and appropriateness of each question, experts were asked to express their opinions on a Likert scale (necessary, useful but not necessary or not necessary). To calculate the CVI of the questions, the formula: “the sum of agreed points for each question / the total number of experts” was used and for CVR, the formula was “CVR = (Ne – N/2)/(N/2), where Ne was the number of agreed points for “essential” and N was the total number of experts” [17].

To perform confirmatory factor analysis of the evaluation criteria, which includes the value of the chi-square index, the normed χ2 measurement index (chi-square ratio of degrees of freedom), adjusted goodness of fit index (AGFI), goodness of fit index (GFI), normal fit index (NFI), incremental fit index (IFI), comparative fit index (CFI), Relative Fit index (RFI), and root mean square error of approximation (RMSEA) were used.

### Internal structure

Data analysis in SPSS version 22 software (IBM Corp., Armonk, NY, USA) and Lisrel version 8.8 software was done. The significance level of tests was considered less than 0.05. Cronbach's alpha with a coefficient equal to or greater than 0.70 (an acceptable level to confirm the internal consistency of the questionnaire) [18] was used to determine the internal consistency of the CLEQ questionnaire.

## Results

### Content validity

The content validity index (CVI) values for the items were between 0.8 and 0.9, and the content validity ratio (CVR) value for the entire questionnaire was 0.9.

**Fig. 1:**
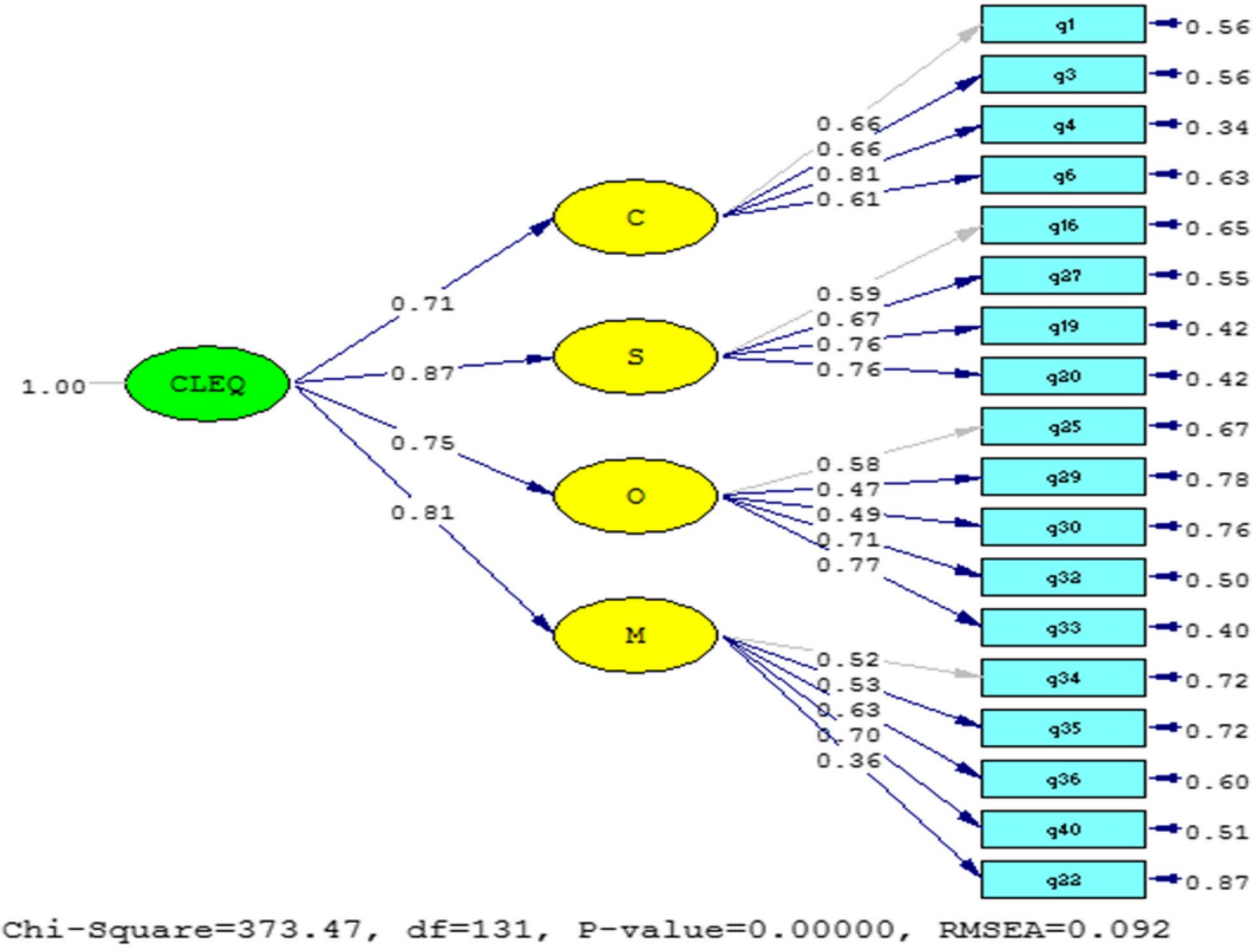
Second-order confirmatory factor analysis model in standard estimation mode

The data included in the arrow connecting the hidden variable (factor) to the observed variable (question) are the same factor loadings. If the factor loading is less than 0.3, the relationship is weak, and the factor loading between 0.3 and 0.6 is acceptable and greater than 0.6 is very desirable [19]. According to the standardized factor loadings, it can be seen that each dimension with a larger factor load has a stronger relationship with the corresponding variable. As shown in the model, all factor loading coefficients for both dimensions and indicators are above 0.3, so all dimensions and indicators are involved in the construction of the mentioned questionnaire (Fig. 1).

### Internal structure

**Fig. 2:**
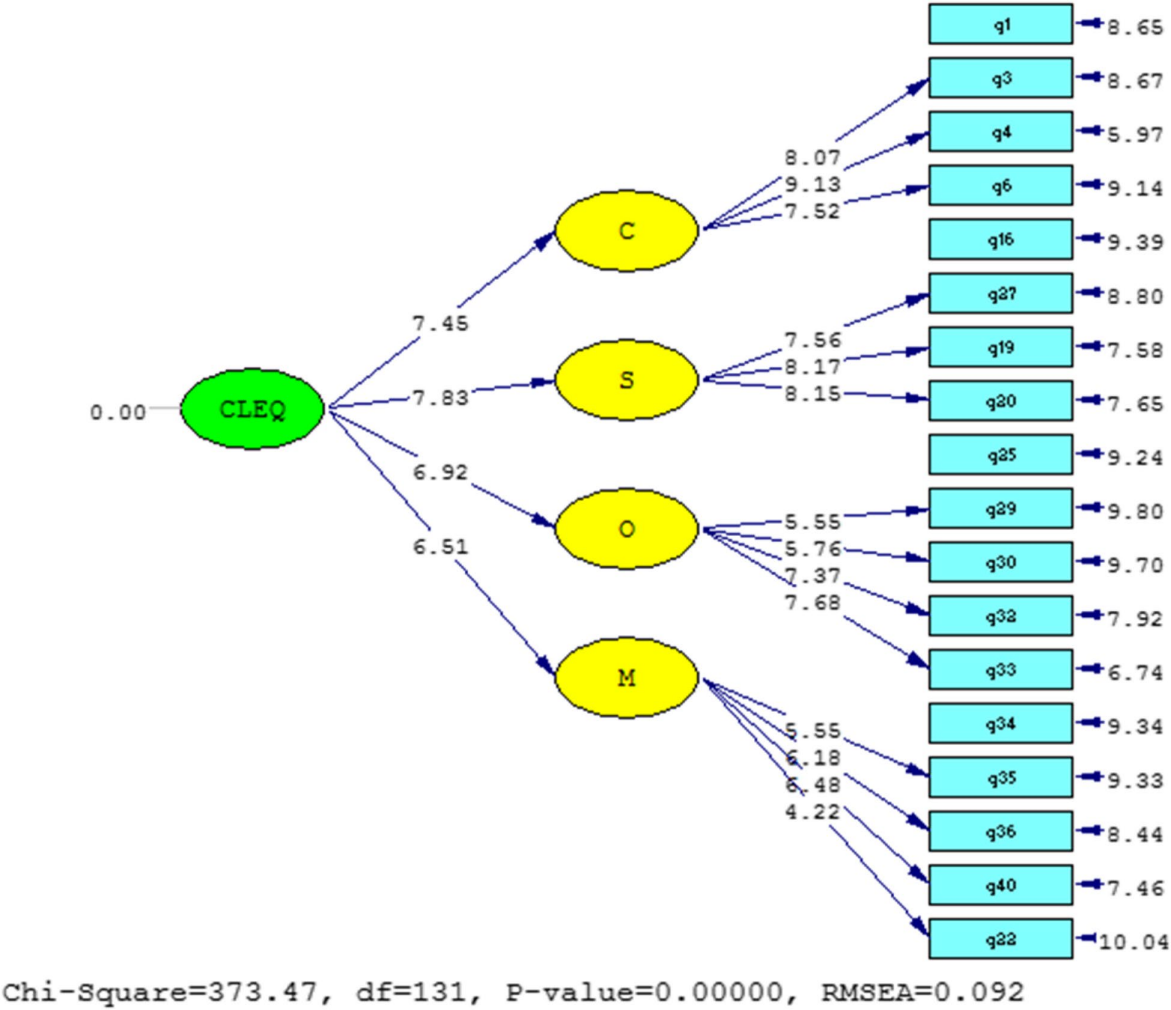
Second-order confirmatory factor analysis model in the significance mode of the coefficients of the dimensions of the questionnaire

Figure 1 shows the significant part of the obtained coefficients and parameters of the model in T-Value mode.If the significant number is greater than 1.96 or less than -1.96, the relationship in the research model will be significant [19]. Figure 2 shows that all relationships are significant.

It is very important to pay attention to the fit of the model in confirmatory factor analysis. Among the fit indices, if CMIN/DF is less than 3, the model has a good fit, which is equal to 2.85 here. In the analysis results of our study, RMSEA is equal to 0.092 while RMSEA values between 0.08 and 0.10 can be considered as a mediocre fit [20]. Other findings from confirmatory factor analysis were evaluated through six criteria, including IFI, RFI, NFI, GFI, AGFI, and CFI, have been shown in Table 1.

**Table 1:** The indicators of fitness of the factor analysis of the CLEQ questionnaire

Structural fitness indicators	**CMIN/DF**	**RMSEA**	**IFI**	**RFI**	**NFI**	**GFI**	**AGFI**	**CFI**
4-dimension structure	2.85	0.092	0.91	0.95	0.90	0.91	0.93	0.95

*CMIN/DF* Chi-square ratio of the degrees of freedom, *RMSEA* Root Mean Square Error of Approximation, *IFI* Incremental Fit Index, *RFI* Relative Fit Index, *NFI* Normed Fit Index, *GFI* Goodness of Fit Index, *AGFI* Adjusted Goodness of Fit Index, *CFI* Comparative Fit Index

The results of Table 1 indicate that all the indicators are reported at the optimal level, the model has a relative fit with the data, and this indicates that the questions are aligned with the theoretical structure and dimensions of the questionnaire.

The reliability of the questionnaire was obtained using Cronbach's alpha coefficient, which is equal to 0.87, which indicates the appropriate reliability of the questionnaire, and the reliability of each dimension is reported in Table 2.

**Table 2:** Cronbach’s alpha, mean and standard deviation of each dimension of the questionnaire

Subscale	Number of Items	Cronbach’s alpha	Mean (SD)
Provision of clinical cases	4	0.77	10.16 (3.75)
Supervision	4	0.79	11.73 (3.99)
Organizing MD-patient encounters	5	0.74	16.85 (4.44)
Student learning motivation	5	0.68	12.28 (3.91)
CLEQ	18	0.87	51 (12.53)

## Discussion

This study aimed to investigate the applicability of the 4-factor model from the original second version of the CLEQ, validated by Alnaami et al., to the Persian translation of the scale. To achieve this, the 18-question version of the CLEQ was translated into Persian language to assess its validity and reliability among medical students at Shiraz University of Medical Sciences.

Persian questionnaire used in the study has good content validity. The content validity index (CVI) values for the items were high, ranging from 0.8 to 0.9, indicating that the items were relevant and representative of the construct being measured. Additionally, the content validity ratio (CVR) value for the entire questionnaire was high at 0.9, indicating that the questionnaire had good overall content validity [20].

The factor loadings provide insight into the strength of the relationship between the latent variable (factor) and the observed variables (questions) [21]. The standardized factor loadings showed that each dimension with a larger factor loading had a stronger relationship with the corresponding variable. All factor loading coefficients for both dimensions and indicators were above 0.3, indicating that all dimensions and indicators contributed to the construction of the questionnaire and were therefore considered in the final analysis. Overall, the findings suggest that the Persian questionnaire used in the study has good content validity and is a reliable tool for measuring the construct of interest.

Internal consistency was done to measure the reliability of the scale. Internal consistency determines how many items in a tool have the same concept or structure. Therefore, it is related to the interrelationship of items within the test [22]. In this approach, a Cronbach's alpha coefficient of 0.7 or higher indicates acceptable reliability of the instrument [23]. In the present study, the reliability coefficient of the CLEQ was found to be 0.87 for the full scale and 0.68 to 0.79 for subscales. While Cronbach's alpha values for the original six-factor model ranged from 0.60 to 0.86 and for the four-factor model by Alnaami et al. ranged from 0.72 to 0.87 the results of two primary studies on the CLEQ questionnaire show that the 4-dimensional questionnaire with 18 questions has better reliability than the 37-question and 6-dimensional questionnaire. The Persian version of the questionnaire also shows acceptable reliability with 18 questions in for dimensions. However, it may be better to examine the Persian version of the 37-question questionnaire.

Table 1 shows that the fitness indicators of the confirmatory factor analysis(CFA) model presented in the Persian version of the CLEQ components have favorable conditions. The researcher used X2/df, GFI, and RMSEA among absolute fit indices, and RFI, NFI, and CFI among other comparative fit indices. The results of these tests showed that X2/df ≤ 3, CFI,RFI, NFI and IFI > 0.9, and RMSEA < 0.1 indicate an acceptable fit [24]. In comparison to Alnaami et al.’s study on the CLEQ questionnaire with different dimensions, the CFA levels in the 4-dimensional questionnaire were found to be in the best state with CFI, RFI, NFI and IFI between 0.865 and 0.951, while the results of our study for these indicators were between 0.90 and 0.95.

Regarding the RMSEA study of Alnaami et al., a good result of 0.052 was obtained, but the results of our study showed an RMSEA index of 0.092. However, considering that the GFI index is higher than 0.9, it seems that the fit of the model is acceptable, and increasing the sample size can improve the RMSEA.

The values of goodness-of-fit statistics indicated that the 4-factor model fits the sample data. However, it may be better to examine the 5-factor and 6-factor models in Persian. Additional studies are necessary to compare the compatibility of the 4-factor model with the 5-factor and 6-factor models for Iranian students. Nonetheless, the correlation between the loading estimates and the dimensions in the path diagram, suggests that the data fit the 4-factor model.

It should be noted that the initial questionnaire was prepared in English and was examined in a country with a native Arabic language, which may limit its generalizability to students at English-speaking universities. Furthermore, comparing the results of the present study in Persian language with the source article reviewed in an Arabic-speaking country may not be generalizable. Therefore, it may be better to examine the initial questionnaire with six dimensions and the final questionnaire with four dimensions at English speaking universities. Another limitation of our study is that we only examined the four-factor model of the CLEQ tool in the Persian language. It would be necessary to explore the applicability of the five-factor and six-factor models in Persian language in order to compare their fit with Iranian data.

## Conclusions

The Persian translation of the 4-factor CLEQ has sufficient validity evidence to measure the transcultural adaptability of clinical education activities by instructors and students. The validity evidence are content, response process and internal structure.

The findings of this study have important implications for medical students in Iran. The Persian version of the CLEQ has been found to have good reliability and validity, indicating that it is a useful tool for assessing clinical learning environments among medical students in Iran. The 4-factor model of the CLEQ was found to be applicable to the Persian translation of the scale, suggesting that it can be used to assess the quality of clinical learning environments in medical schools in Iran.

The results of this study can be used to identify areas of strength and weakness in clinical learning environments and to develop interventions to improve the quality of clinical education for medical students in Iran. Overall, the findings of this study can be used to improve the quality of clinical education for medical students in Iran and to ensure that they are well-prepared for their future roles as healthcare professionals.

## Data Availability

The datasets used and analyzed during the current study are available from the corresponding author on request. The data are not publicly available due to privacy or ethical restrictions.

## Abbreviations

CLE: Clinical Learning Environment
CLEQ: Clinical Learning Evaluation Questionnaire
PHEEM: Postgraduate Hospital Educational Environment Measure
DREEM: Dundee Ready Education Environment Measure
CVR: Content Validity Ratio
CVI: Content Validity Index
CFA: Confrmatory factor analysis
RMSEA: Root Mean Square Error of Approximation
GFI: Goodness of Fit Index
AGFI: Adjusted Goodness of Fit Index
NFI: Normed Fit Index
CFI: Comparative Fit
IFI: Incremental Fit Index
